# The implementation of academic detailing and its effectiveness on appropriate prescribing of pain relief medication: a real-world cluster randomized trial in Belgian general practices

**DOI:** 10.1186/s13012-017-0703-8

**Published:** 2018-01-10

**Authors:** Robin Bruyndonckx, Veronique Verhoeven, Sibyl Anthierens, Koen Cornelis, Katelijne Ackaert, Birgit Gielen, Samuel Coenen

**Affiliations:** 1 Laboratory of Medical Microbiology, Vaccine & Infectious Disease Institute (VAXINFECTIO), University of Antwerp, Antwerp, Belgium; 2 Interuniversity Institute for Biostatistics and statistical Bioinformatics (I-BIOSTAT), University of Hasselt, Agoralaan building D, 3590 Diepenbeek, Hasselt, Belgium; 3 Department of Primary and Interdisciplinary Care (ELIZA), University of Antwerp, Antwerp, Belgium; 4 Intermutualistic Agency (IMA-AIM), Brussels, Belgium; 5 Department of Epidemiology and Social Medicine (ESOC), University of Antwerp, Antwerp, Belgium

**Keywords:** Continuing medical education, Educational outreach visits, Nonsteroidal anti-inflammatory drugs, Primary care, Prescribing behaviour, Interrupted time series analysis

## Abstract

**Background:**

In Belgium, the debate about the effect of the national academic detailing service (ADS) on prescribing quality in general practice is ongoing. In order to evaluate both the implementation strategies of the ADS and its effectiveness on appropriate prescribing of pain relief medication, we conducted a real-world cluster randomized controlled trial (cRCT).

**Methods:**

In a pragmatic cRCT, all Belgian general practices previously visited by Farmaka were assessed for eligibility and randomized. Only practices randomized to the intervention group were invited for an academic detailing visit on appropriate prescribing of pain relief medication. GPs were unaware of the study, ensuring the production of real-world evidence but were given the option to opt out from the analysis. An objective outcome assessment was obtained using routinely collected reimbursement data. Primary outcomes were the proportion of patients reimbursed for an analgesic or NSAID, the defined daily dose of paracetamol per patient per month, the proportion of patients reimbursed for a recommended NSAID among those reimbursed for any NSAID and the proportion of patients reimbursed for both an NSAID and a proton pump inhibitor among those reimbursed for an NSAID. The impact of practice, GP and academic detailer characteristics were also assessed.

**Results:**

Three thousand five hundred twenty-nine general practices (4530 GPs) were eligible and randomized. One thousand six hundred ninety-eight practices (2171 GPs) in the intervention group and one thousand seven hundred three (2163 GPs) in the control group were included in the analysis. The intervention had a significant impact on the proportion of patients reimbursed for a recommended NSAID among those reimbursed for any NSAID (increase in odds (95% CI): 19% (10–29%)). A clear impact on other outcomes could not be detected. Additionally, we showed that the characteristics of the academic detailers might impact the effectiveness of the visit.

**Conclusions:**

National implementation of academic detailing in Belgian general practices provided by Farmaka significantly improved the proportion of recommended NSAIDs prescribed by GPs, but not other outcomes related to appropriate prescribing of pain relief medication.

**Trial registration:**

NCT01761864. Registered 2 January 2013.

**Electronic supplementary material:**

The online version of this article (10.1186/s13012-017-0703-8) contains supplementary material, which is available to authorized users.

## Background

Several continuing medical education (CME) initiatives aim to support general practitioners (GPs) to cope with the ever increasing amount of novel professional information. In Western countries, CME activities rely heavily on support from the pharmaceutical industry, introducing bias in both topic selection and information provided [1]. Most CME strategies (e.g. conferences) have mixed effects, but academic detailing (AD) is especially effective for improving the quality of prescribing in general practice [2]. A recent synthesis of systematic reviews showed that CME had a small but significant impact on physician performance (6%) and patient health outcomes (3%). [3]. A Cochrane review of randomized controlled trials (RCTs) showed that AD improved quality of care, with the effect on prescribing quality being small (3–6.5%) but larger than that of other strategies [4]. Additionally, AD has been shown to be feasible with a majority of GPs who wished to receive future visits [5].

Farmaka (www.farmaka.be) is an independent Belgian drug information centre operating nationwide to improve rational prescribing and is funded by the Federal Agency for Medicines and Health Products (FAMHP; www.famhp.be/en/famhp). The effect of its academic detailing service (ADS) on improved prescribing has been shown in two small RCTs [6, 7]. Another study however could not demonstrate its effectiveness [8]. The latter study was ordered by FAMHP because it nearly doubled its yearly investment in its ADS in 2009. This limited evaluation was conducted by the Belgian health care knowledge centre (KCE) on AD visits covering dementia and diabetes and did not allow to draw valid conclusions.

Therefore, the FAMHP ordered a new evaluation of its ADS. Instead of conducting another traditional trial, we chose to produce real-world evidence on both the effectiveness and implementation strategies of the ADS by also assessing the impact of practice, GP and academic detailer characteristics on ADS’ effectiveness and to complement this trial with a process evaluation [9, 10].

The academic detailers’ visits are structured around prescribing for common medical conditions in general practice (ranging from acute cough to anxiety disorders) and are offered to GPs on a voluntarily basis. In line with the planning of the ADS and the recommendations by Borgermans et al., we focused on the visits in 2013 on appropriate use of pain relief medication (analgesics and NSAIDs) for chronic pain in osteoarthritis which is a common condition in which the GP plays an important role (rather than a specialist) and for which a great impact could be expected given the room for improvement in osteoarthritis care. This topic was handled by Farmaka because osteoarthritis is a condition that occurs more and more frequently, due to the ageing population, and for which high doses of non-steroidal anti-inflammatory drugs (NSAIDs) are prescribed. Chronic use of NSAIDs however is associated with gastrointestinal (GI) adverse effects, such as upper GI bleeding and perforation [11–17].

In this pragmatic cluster RCT (cRCT), we will evaluate real-world evidence on the implementation of academic detailing by Farmaka in Belgian general practices by assessing its effectiveness on appropriate prescribing of pain relief medication for chronic pain relief in osteoarthritis and factors that modify its effectiveness.

## Methods

### Study population and trial design

**Fig. 1:**
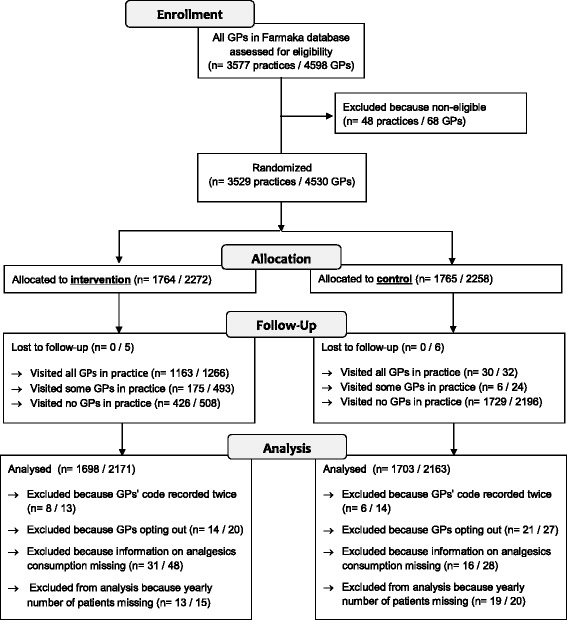
Flow diagram of enrolled, allocated and analysed practices

Belgian general practices that were visited before by a Farmaka academic detailer were assessed for eligibility and those still eligible (i.e. still practicing) were allocated at random in a 1:1 ratio to an intervention group, which was invited for a visit on chronic pain relief in osteoarthritis by an academic detailer, or to a control group that did not receive a visit on this topic (Fig. 1). A cluster randomization, randomizing practices rather than individual GPs, was chosen to minimize contamination between GPs in practices with more than one GP. Randomization was performed in permuted blocks of two after stratification of practices according to academic detailer (17), the number of visits received in the past (ranging from 1 to 16) and the type of practice (including one, two or more than two GPs) in order to optimize comparability between intervention and control practices. For the same reason, practices were sorted within their strata by descending GP identification code (GP ID) of the GP with the highest GP ID reflecting an earlier registration of that GP by the Belgian Government, before randomization. Within the randomized blocks, i.e. pairs of intervention and control practices, the date of visit for the practice in the intervention group could be used for the control group as well, ensuring a comparable spread of dates of visit in the control group. Whenever a practice in the intervention group was not visited or the date of visit was not noted, the latest date of visit was used. To evaluate the influence of this choice on the results, a sensitivity analysis, excluding the practices for which no visitation date was noted, was conducted. Group allocation was concealed for all randomized GPs until the end of the outcome assessment. The outcomes were assessed objectively, by analysing routinely collected reimbursement data. Before data became available for analysis, all randomized GPs were informed of the study through written communication (in November 2014) and were given the option to be excluded from the analysis (opt out) by written notice before 23 December 2014. Blinding of academic detailers was not possible due to the nature of the intervention. The trial was approved by the ethics committee of the University of Antwerp/Antwerp University Hospital (B300201317018) and registered with clinicaltrials.gov (NCT01761864).

### Intervention

**Fig. 2:**
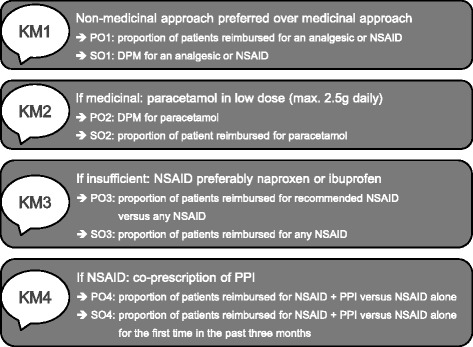
Overview of key messages and outcomes included in the arthrosis information. Package. KM key message, PO primary outcome, SO secondary outcome, DPM defined daily dose (DDD) per patient per month, NSAID non-steroidal anti-inflammatory drugs, PPI proton pomp inhibitor

Practices in the intervention group were offered a (free) 15- to 20-min visit by an academic detailer, who provided four comprehensible key messages (KM) on prescription of pain relief medication in patients with osteoarthritis (Fig. 2). First, a non-medicinal approach should be preferred [KM1]. Second, if a medicinal approach is needed, paracetamol is the first choice and should not be dosed too high (max. 2.5 g daily) [KM2]. Third, NSAIDs could be used when response to paracetamol is insufficient. In that case, ibuprofen or naproxen is recommended due to their safety profile and cost-benefit ratio [12–14] [KM3]. Fourth, when prescribing NSAIDs, gastroprotective agents, such as proton pump inhibitors (PPIs), should be co-prescribed to patients with gastrointestinal risk factors (e.g. aged 65 or above, presence of comorbidity or history of ulcer formation) to reduce the risk of complications [15–17] [KM4]. The information package used by the academic detailers can be found in Additional file 1.

Practices in the control group were not offered a visit.

### Data characteristics

Data on the characteristics of the eligible general practices used for the randomization (unique identification codes for the visiting academic detailer (AD ID) and the practice (practice ID), GP ID, the number of previous visits by a Farmaka academic detailer and the practice type) and on the characteristics of the academic detailers (education and experience) was provided by Farmaka that collects and checks these routine data as part of its ADS. Additional data on the characteristics of the eligible general practices (GP ID, number of included GPs in the practice the GP belongs to, yearly number of assigned patients to the practice the GP belongs to, region and province) and on the reimbursement of selected pain relief medication they prescribed to patients aged 60 and over between 6 months before and 6 months after the intervention date (Anatomic Therapeutic Chemical (ATC) code N02BE01 (paracetamol), N02 (general analgesics), M01AE01 (ibuprofen), M01AE02 (naproxen), M01A (NSAIDs) and A02BC (PPIs)) was provided by the Intermutualistic Agency (IMA; www.aim-ima.be), collecting these data from the seven health insurance companies in Belgium for health care research. All data were linked through the GP ID and aggregated at the level of the general practice. Whenever there was uncertainty about which academic detailer provided the visit, the code (AD ID) of the academic detailer to which the practice was randomized was used in the analysis.

### Outcomes definition

Four primary outcomes (POs) and four secondary outcomes (SOs) (all at the level of the general practice) were extracted from the key messages included in the osteoarthritis information package (Fig. 2).

#### Primary outcomes

The first PO, assessing the effectiveness of KM1, was defined as the proportion of patients reimbursed for an analgesic of NSAID (PO1). It was calculated as the monthly number of patients reimbursed for a drug with ATC code N02 or M01A, divided by the number of patients assigned yearly (at practice level). If KM1 was conveyed successfully, we expect PO1 to decrease by prescribing proportionally less patients with an analgesic. The second PO, assessing the effectiveness of KM2, was defined as the defined daily dose (DDD) per patient per month (DPM) for paracetamol (PO2). It was calculated as the monthly DDD for drugs with ATC code N02BE01 divided by the monthly number of patients reimbursed for a drug with ATC code N02BE01 (at practice level). If KM2 was conveyed successfully, we expect the DPM of paracetamol, and hence PO2, to decrease. The third PO, assessing the effectiveness of KM3, was defined as the proportion of patients reimbursed for a recommended NSAID among those reimbursed for any NSAID (PO3). It was calculated as the monthly number of patients reimbursed for a drug with ATC code M01AE01 or M01AE02, divided by the monthly number of patients reimbursed for a drug with ATC code M01A (at practice level). If KM3 was conveyed successfully, we expect PO3 to increase by prescribing proportionally more recommended NSAIDs. The fourth PO, assessing KM4, was defined as the proportion of patients reimbursed for both an NSAID and a PPI among those reimbursed for an NSAID (PO4). It was calculated as the monthly number of patients reimbursed for a drug with ATC code M01A and a drug with ATC code A02BC, divided by the monthly number of patients reimbursed for a drug with ATC code M01A (at practice level). If KM4 was conveyed successfully, we expect PO4 to increase by prescribing proportionally more patients with both an NSAID and a PPI.

#### Secondary outcomes

In order to obtain a complete picture of the effectiveness of the implementation of KM1, the consumption of analgesics and NSAID was also assessed using the DPM for analgesics (SO1). The DPM was calculated as the monthly DDD for drugs with ATC code N02 or M01A, divided by the monthly number of patients reimbursed for a drug with ATC code N02 or M01A (at practice level). If KM1 was conveyed successfully, we expect the DPM of analgesics, and hence SO1, to decrease. Because KM2 also implied that paracetamol should be used as the first choice, the proportion of patients reimbursed for paracetamol was studied (S02). This outcome was calculated as the number of patients reimbursed for a drug with ATC code N02BE01, divided by the yearly number of assigned patients (at practice level). If KM2 was conveyed successfully, we expect SO2 to increase by prescribing proportionally more patients with paracetamol. Because KM3 also implied that NSAIDs should be used as a second medicinal approach, the proportion of patients reimbursed for any NSAID was studied (SO3). This outcome was calculated as the number of patients reimbursed for a drug with ATC code M01A, divided by the yearly number of assigned patients (at practice level). If KM3 was conveyed successfully, we expect SO3 to decrease by prescribing proportionally less patients with paracetamol. Because we believe that introducing an additional product (being PPI) to patients already using NSAID would be harder than introducing two new products together (being NSAID + PPI), the proportion of patients reimbursed for both an NSAID and a PPI among those reimbursed for an NSAID for the first time in 3 months was studied (SO4). This outcome was calculated as the monthly number of patients reimbursed for a drug with ATC code M01A and a drug with ATC code A02BC for the first time in the past 3 months, divided by the monthly number of patients reimbursed for a drug with ATC code M01A for the first time in the past 3 months (at practice level). If KM4 was conveyed successfully, we expect SO4 to increase by prescribing proportionally more patients with an NSAID and a PPI when receiving an NSAID for the first time in 3 months.

#### Power calculations

At the 5% significance level, this cRCT with a maximum sample size of 3500 at the level of the practice has more than 80% power to detect absolute differences between the intervention groups ≥ 5% around the most conservative proportion of 50% (e.g. 47.5 vs 52.5%) and standardized differences in means of 0.1 (e.g. standardized difference in mean DPM of 1 for a standard deviation of 10).

### Analysis settings

**Fig. 3:**
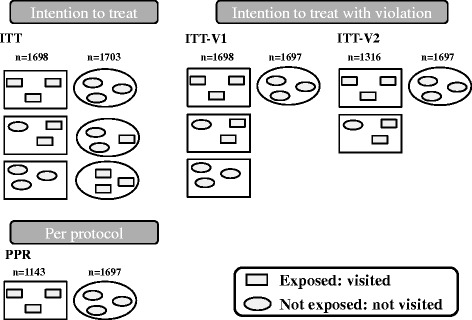
Schematic representation of different analysing approaches. Frame, identification of practice after randomization; symbol, identification of practice after data collection; *n*, number of practices

The primary analysis was an unadjusted intention to treat (ITT) analysis (Fig. 3, top left panel) in which all practices were analysed in the groups to which they were randomized. As randomization was performed at the level of the practice, in theory, all GPs within one practice should either have or have not been visited. In reality, however, among the control practices that should not have been visited, there were practices with no visits, practices in which some GPs were visited (partially non-adherent control, e.g. GP transferred from intervention to control practice after randomization) and practices in which all GPs were visited (fully non-adherent control, e.g. GP from a solo practice transferred to an intervention practice after randomization). In addition, among the intervention practices that should have been visited, there were practices in which all GPs were visited, practices in which only some GPs were visited (partially non-adherent intervention, e.g. one GP absent on the day of the visit) and practices in which no GP was visited (fully non-adherent intervention, e.g. no appointment could be made). In order to assess the robustness of the findings from the ITT analysis, three additional analysis settings were created (Fig. 3). The ITT analysis setting with violation (ITT-V1) was obtained by leaving out all non-adherent control practices. The ITT-V2 analysis setting was obtained by also leaving out the fully non-adherent intervention practices. The per protocol (PPR) analysis setting was obtained by also leaving out the partially non-adherent intervention practices.

### Statistical analysis

The primary analysis compared the intervention and control practices. Because we anticipated that the intervention might cause a shift in prescribing behaviour, which might fade over time, we used an interrupted time series approach [18, 19]. Because monthly observations were clustered by practice, and practices were clustered within provinces, we corrected the interrupted time series model for clustering by province [20–22]. Because some of the explanatory variables were varying with time (e.g. the number of GPs which might differ over a period of 12 months), we used an independent working correlation in order to obtain unbiased results [23]. Note that, even if this correlation structure does not match the true structure, obtained estimates and robust standard errors will be statistically consistent [20].

The model we used can be presented as follows:

$$ g\left({Y}_i\right)={\beta}_0+{\beta}_1 Tr+{\beta}_2{T}_i+{\beta}_3{X}_i+{\beta}_4{Ta}_i+{\beta}_5 Tr.{T}_i+{\beta}_6 Tr.{X}_i+{\beta}_7 Tr.{Ta}_i $$

where *Y*_*i*_ represents the outcome at time point *i*, with *i* ranging from 1 (at 6 calendar months before the intervention) to 12 (at 6 calendar months after the intervention), *g*() represents the link function (an identity link is used for PO2 and SO1, a logit link is used for the other outcomes), *Tr* is a dummy variable to indicate the intervention group (intervention: 1, control: 0), *T*_*i*_ is a continuous variable (1–12) indicating the time in months (from 6 months before to 6 months after the intervention), *X*_*i*_ is a dummy variable indicating pre- or post-intervention period (0: pre, 1: post), *Ta*_*i*_ is a continuous variable (1–6) indicating the time after intervention in months. *β*_0_ represents the outcome in the control group at baseline (i.e. 6 months before intervention), *β*_1_ represents the difference in outcome between the intervention and the control group at baseline, *β*_2_ represents the trend of the outcome in the control group before the intervention, *β*_3_ represents the step change in the outcome for the control group immediately after the intervention, *β*_4_ represents the change in the trend of the outcome after the intervention in the control group, *β*_5_ represents the difference in trend of the outcome between the intervention and the control group before intervention, *β*_6_ represents the difference in step change between the intervention and the control group immediately after the intervention (the step change) and *β*_7_represents the difference in the change in trend of the outcome between the intervention and the control group after intervention (the change in trend).

Predicted proportions (or DPMs) for analyses in which the intervention had a consistent and significant impact were obtained using the model’s parameter estimates. Confidence bounds around the estimates were obtained using the delta method [24].

#### Exploratory subgroup analyses

We pre-specified the assessment of effect modification by the number of previously received visits and the characteristics of the academic detailer (a physician or not). In addition, we looked at the outcomes in subgroups defined by experience of the academic detailer (at least 40 years of age or not), region of the practice (Flanders versus Wallonia, with Brussels included in Wallonia) and number of GPs in the practice. For the last two variables, we examined the homogeneity of the step change and the change in trend after the intervention across different subgroups by including interaction terms between time components, intervention and subgroup (while correcting for baseline differences). For the first three variables, which could by design only impact the intervention group, we examined the homogeneity of the step change and the change in trend after the intervention across the subgroups by changing the model described above to adjust for subgroup effects rather than intervention effects and excluding all control practices from the analysis.

#### Sensitivity analyses

Because the date on which the academic detailer visited the practice was not reported in approximately 20% of included practices, we conducted a sensitivity analyses in which these practices were excluded (i.e. 22.29% of practices covering 14.10% of observations divided equally between the intervention and the control groups).

## Results

Figure 1 shows the number of practices (and GPs) who were enrolled, allocated, followed up and analysed. Of the 3577 practices (4598 GPs) that were assessed for eligibility, 48 practices (68 GPs) were excluded because they were not professionally active anymore (e.g. retired or deceased). The remaining 3529 practices (4530 GPs) were randomized: 1764 practices (2272 GPs) were assigned to the intervention group and were scheduled to receive a visit by an academic detailer between 10 February and 22 November 2013, and 1765 practices (2258 GPs) were assigned to the control group. There were no practices but 11 GPs for which we could not assess whether they had been visited or not (5 GPs in intervention group, 6 GPs in control group). Before analysing the results, we excluded 14 practices (8 (13 GPs) in intervention group, 6 (14 GPs) in control group) because their GP code occurred twice, 35 practices (14 (20 GPs) in intervention group, 21 (27 GPs) in control group) because GPs chose to opt out, 47 practices (31 (48 GPs) in intervention group, 16 (28 GPs) in control group) because we could not obtain any information on consumption of pain relief medication (most likely these GPs stopped practicing) and 32 practices (13 (15 GPs) in intervention group, 19 (20 GPs) in control group) because we could not obtain the number of patients visiting yearly. We analysed 3401 practices (1698 in intervention group, 1703 in control group) including 4334 GPs (2171 in intervention group, 2163 in control group). Baseline characteristics were similar in the intervention and the control groups (Table 1).

**Table 1:** Characteristics of analysed practices

	Intervention	Control
Practices (*N* (%))*	1698	1703
Region		
- Flanders	912 (53.7)	919 (54.0)
- Wallonia and Brussels	786 (46.3)	784 (46.0)
Province		
- Antwerp	225 (13.3)	222 (13.0)
- East Flanders	331 (19.5)	334 (19.6)
- Flemish Brabant	82 (4.8)	88 (5.2)
- Limburg	0 (0)	0 (0)
- West Flanders	278 (16.4)	276 (16.2)
- Hainaut	140 (8.2)	137 (8.0)
- Liege	198 (11.7)	191 (11.2)
- Luxembourg	82 (4.8)	87 (5.1)
- Namur	125 (7.4)	115 (6.8)
- Walloon Brabant	98 (5.8)	105 (6.2)
- Brussel	143 (8.4)	149 (8.7)
Academic detailer (*n* = 22)		
- ID: 47	104 (6.1)	109 (6.4)
- ID: 59	108 (6.4)	105 (6.2)
- ID: 64	48 (2.8)	46 (2.7)
- ID: 72	26 (1.5)	77 (4.5)
- ID: 77	112 (6.6)	114 (6.7)
- ID: 79	83 (4.9)	85 (5.0)
- ID: 81	103 (6.1)	100 (5.9)
- ID: 82	79 (4.7)	67 (3.9)
- ID: 86	82 (4.8)	79 (4.6)
- ID: 92	151 (8.9)	154 (9.0)
- ID: 96	150 (8.8)	157 (9.2)
- ID: 97	122 (7.2)	122 (7.2)
- ID: 99	84 (4.9)	83 (4.9)
- ID: 100	57 (3.4)	61 (3.6)
- ID: 101	60 (3.5)	60 (3.5)
- ID: 105	163 (9.6)	163 (9.6)
- ID: 114	128 (7.5)	84 (4.9)
- Academic detailers replacing randomized academic detailers (ID: 3, 75, 88, 95, 115)	61 (3.6)	40 (2.3)
Age of academic detailer (*n* = 22)		
- < 40 years (*n* = 10)	1018 (60.0)	997 (58.5)
- ≥ 40 years (*n* = 7)	609 (35.9)	593 (34.8)
- Age unknown at time of analysis (*n* = 5)	87 (5.1)	117 (6.9)
Education of academic detailer (*n* = 22)		
- Physician (*n* = 6)	457 (26.9)	459 (27.0)
- Non-physician (*n* = 15)	1221 (71.9)	1167 (68.5)
- Training unknown at time of analysis (*n* = 1)	26 (1.5)	77 (4.5)
Number of previous visits (to at least one general practitioner in the practice)		
- 1	268 (15.8)	276 (16.2)
- 2	229 (13.5)	225 (13.2)
- 3	222 (13.1)	210 (12.3)
- 4	203 (12.0)	197 (11.6)
- 5	214 (12.6)	221 (13.0)
- 6	202 (11.9)	200 (11.7)
- 7	183 (10.8)	190 (11.2)
- 8	150 (8.8)	151 (8.9)
- 9	124 (7.3)	118 (6.9)
- 10	89 (5.2)	87 (5.1)
- > 10	148 (8.7)	136 (8.0)
Number of general practitioners in the practice that were visited by Farmaka before		
- 1	1397 (82.3)	1399 (82.1)
- 2	203 (12.0)	200 (11.7)
- 3 or more	98 (5.8)	104 (6.2)
Number of general practitioners	2171	2163
Mean (SD) number of general practitioners per practice	1 (1)	1 (1)
Mean (SD) number of patients (≥ 60 years) per practice	377 (240)	373 (242)
Proportion of reimbursed prescriptions under study		
Analgesics (N02)	37.9	37.9
Paracetamol (N02BE01)	7.5	7.7
Proton pump inhibitor (A02BC)	33.1	33.2
Nonsteroidal anti-inflammatory drug (NSAID; M01A) combined with proton pump inhibitor (A02BC)	5.4	5.3
Nonsteroidal anti-inflammatory drugs (NSAIDS; M01A)	29.0	28.8
Ibuprofen (M01AE01)	6.8	6.7
Naproxen (M01AE02)	1.7	1.6
Recommended NSAIDS (M01AE01 or M01AE02) among all NSAIDS	29.3	28.8
Tilidine (N02AX01)	2.6	2.6
Tramadol (N02AX02)	9.9	9.6

*Some practices are counted double in this table, e.g. practices in which general practitioners have an address in different provinces, are visited by different academic detailers, had a different number of previous visits

### Assessment of effectiveness using primary outcomes

**Fig. 4:**
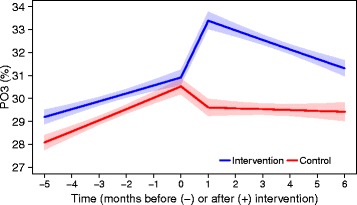
Predicted proportions (95% confidence bounds) for PO3 in an intention to treat analysis. PO3, proportion of patients reimbursed for a recommended nonsteroidal anti-inflammatory drug (NSAID) among those reimbursed for any NSAID

The primary analyses (ITT) show that, in general, the odds of being reimbursed for an analgesic or NSAID was decreasing significantly over time. In the intervention group, this decrease was stronger after the intervention (Table 2). The DPM for paracetamol was increasing significantly over time, without a clear impact of the intervention. The odds of being reimbursed for a recommended NSAID when reimbursed for any NSAID was increasing significantly over time. At the time of the intervention, there was a significant upward shift for the practices that encountered an academic detailer compared to practices in the control group. After this shift, the odds slowly, but significantly, decreased again (both in the intervention and the control groups) (Fig. 4). The odds of being reimbursed for an NSAID and a PPI when reimbursed for an NSAID did not change significantly. Because, for some outcomes, different conclusions were drawn from the other three settings (ITT-V1, ITT-V2 and PPR; results in Appendix), we focus on significant results that are consistent over the four settings (underlined).

**Table 2:** Impact of academic detailing visits on primary outcomes in an intention to treat analysis

	Step change (*β*_6_) [99% Wald CI]	Change in trend (*β*_7_) [99% Wald CI]
Odds of being reimbursed for an analgesic or NSAID, in intervention compared to control group (PO1)	0.9980 [0.9834; 1.0128]	0.9889 [0.9767; 1.0013]
Average defined daily dose of paracetamol per patient reimbursed for paracetamol per month, in intervention compared to control group (PO2)	− 0.3287 [− 1.0491; 0.3917]	0.0386 [− 0.1973; 0.2745]
Odds of being reimbursed for a recommended NSAID when reimbursed for any NSAID, in intervention compared to control group (PO3)	1.1903*[1.0757; 1.3171]	0.9901 [0.9719; 1.0086]
Odds of being reimbursed for an NSAID and a PPI when reimbursed for an NSAID, in intervention compared to control group (PO4)	1.0217 [0.9243; 1.1294]	0.9926 [0.9685; 1.0173]

*Step change* difference in step change between the intervention and control group immediately after the intervention; *change in trend* difference between the change in trend between the intervention and control group after the intervention; *CI* confidence interval, *NSAID* nonsteroidal anti-inflammatory drug, *PPI* proton pump inhibitor; *p value < 0.01; *underlined* < 0.01 in all four analyses (ITT, ITT-V1, ITT-V2 and PPR)

### Assessment of effectiveness using secondary outcomes

The analyses show that the DPM for pain relief medication did not change significantly. The proportion of patients reimbursed for paracetamol decreased significantly after the intervention, without a clear shift at the time of intervention (Table 3). The overall proportion of patients reimbursed for any NSAID was decreasing significantly over time, without a clear impact of the intervention. The proportion of patients reimbursed for both an NSAID and a PPI among those reimbursed for an NSAID for the first time in 3 months did not change significantly.

**Table 3:** Impact of academic detailing visits on secondary outcomes in an intention to treat analysis

	Step change (*β*_6_) [99% Wald CI]	Change in trend (*β*_7_) [99% Wald CI]
Average defined daily dose of analgesic and NSAIDs per patient reimbursed for an analgesic or NSAID per month, in intervention compared to control group (SO1)	0.0630 [− 0.2600; 0.3861]	0.0470 [− 0.0384; 0.1323]
Odds of being reimbursed for paracetamol, in intervention compared to control group (SO2)	1.0072 [0.9863; 1.0286]	0.9874 [0.9742; 1.0009]
Odds of being reimbursed for an NSAID, in intervention compared to control group (SO3)	1.0087 [0.9913; 1.0263]	0.9913 [0.9773; 1.0056]
Odds of being reimbursed for both an NSAID and a PPI when reimbursed for an NSAID for the first time in 3 months, in intervention compared to control group (SO4)	1.0450 [0.9520; 1.1472]	0.9907 [0.9434; 1.0404]

*Step change* difference in step change between the intervention and control group immediately after the intervention; *change in trend* difference between the change in trend between the intervention and control group after the intervention; *CI* confidence interval; *CI* confidence interval; *NSAID* nonsteroidal anti-inflammatory drug; *PPI* proton pump inhibitor; *p value < 0.05; underlined, < 0.05 in all four analyses (ITT, ITT-V1, ITT-V2 and PPR)

### Exploratory subgroup analyses

All characteristics under study had a significant impact on the intervention (Tables 4 and 5). Because different conclusions were reached in the four settings in this exploratory analysis (ITT, ITT-V1, ITT-V2 and PPR), we will focus on significant results that are consistent over the four settings (underlined).

**Table 4:** Covariates’ effect on the impact of the intervention on primary outcomes in an intention to treat analysis

	PO1: odds ratio [95% Wald CI]	PO2: estimate [95% Wald CI]	PO3: odds ratio [95% Wald CI]	PO4: odds ratio [95% Wald CI]
Region (Flanders)				
Step change	1.0191 [0.9937; 1.0452]	1.3193** [0.4784; 2.1603]	0.979 [0.8346; 1.1483]	1.0559 [0.8941; 1.2470]
Change in trend	0.9970 [0.9791; 1.0153]	− 0.0647 [− 0.4576; 0.3283]	0.9932 [0.9540; 1.0340]	0.9987 [0.9554; 10,439]
Number of general practitioners visited by Farmaka before in the practice				
Step change	1.0062 [0.9893; 1.0235]	0.6101** [0.1596; 1.0606]	0.9726 [0.9364; 1.0102]	0.9977 [0.9213; 1.0805]
Change in trend	0.9922 [0.9843; 1.0001]	− 0.0514 [− 0.1801; 0.0774]	0.9944 [0.9826; 1.0064]	0.9925 [0.9749; 1.0105]
Number of previously received visits				
Step change	1.0002 [0.9977; 1.0027]	− 0.0639 [− 0.2455; 0.1177]	1.0095* [1.0019; 1.0172]	1.0056 [0.9887; 1.0229]
Change in trend	1.0010 [0.9998; 1.0022]	0.0243 [− 0.0166; 0.0653]	0.9993 [0.9972; 1.0014]	0.9978 [0.9953; 1.0004]
Age of academic detailer ≥ 40 years)				
Step change	0.9741 [0.9482; 1.0007]	− 0.8419** [− 1.3166; − 0.3673]	1.0570 [0.9736; 1.1475]	0.9391 [0.8559; 1.0305]
Change in trend	1.0067 [0.9901; 1.0235]	0.2232 [− 0.1231; 0.5696]	0.9922 [0.9691; 1.0159]	1.0087 [0.9930; 1.0247]
Academic detailer physician				
Step change	0.9698 [0.9337; 1.0072]	− 0.7183 [− 1.6650; 0.2284]	1.0786* [1.0038; 1.159]	0.9706 [0.8948; 1.0528]
Change in trend	1.0040 [0.9843; 1.0242]	0.2245 [− 0.0665; 0.5155]	1.0075 [0.9923; 1.0229]	1.0267** [1.0075; 1.0463]

*Step change* difference in step change between the intervention and control group immediately after the intervention; *change in trend* difference between the change in trend between the intervention and control group after the intervention; *PO1* proportion of patients reimbursed for an analgesic; *PO2* average defined daily dose of paracetamol per patient reimbursed for paracetamol per month; *PO3* proportion of patients reimbursed for a recommended nonsteroidal anti-inflammatory drug (NSAID) among those reimbursed for any NSAID; *PO4* proportion of patients reimbursed for an NSAID and a proton-pump inhibitor among those reimbursed for an NSAID; *p value < 0.05; **p value < 0.01; *underlined* < 0.05 in all four analyses (ITT, ITT-V1, ITT-V2 and PPR)

**Table 5:** Covariates’ effect on the impact of the intervention on secondary outcomes in an intention to treat analysis

	SO1: estimate [95% Wald CI]	SO2: odds ratio [95% Wald CI]	SO3: odds ratio [95% Wald CI]	SO4: odds ratio [95% Wald CI]
Region (Flanders)				
Step change	− 0.1550 [− 0.7237; 0.4138]	1.0049 [0.9663–1.0450]	1.0064 [0.9696–1.0445]	0.9705 [0.8587–1.0968]
Change in trend	− 0.1711** [− 0.2866; − 0.0557]	0.9860 [0.9695–1.0026]	1.0076 [0.9900–1.0256]	1.0100 [0.9347–1.0914]
Number of general practitioners visited by Farmaka before in the practice				
Step change	− 0.0553 [− 0.2427; 0.1320]	1.0229* [1.0013–1.0451]	1.0156 [0.9853–1.0469]	0.9844 [0.8718–1.1115]
Change in trend	− 0.0024 [− 0.0730; 0.0683]	1.0014 [0.9910–1.0119]	0.9905* [0.9825–0.9986]	0.9865 [0.9600–1.0137]
Number of previously received visits				
Step change	− 0.0057[− 0.0640; 0.0526]	0.9994 [0.9962–1.0026]	0.9989 [0.9951–1.0027]	1.0059 [0.9912–1.0209]
Change in trend	0.0079[− 0.0055; 0.0213]	0.9999 [0.9973–1.0025]	1.0016** [1.0004–1.0028]	0.9975 [0.9923–1.0028]
Age of academic detailer (≥ 40 years)				
Step change	− 0.2413 [− 0.5875; 0.1050]	0.9791 [0.9183–1.0440]	0.9627** [0.9357–0.9906]	0.9505 [0.8012–1.1269]
Change in trend	0.0991 [− 0.0075; 0.2056]	1.0149 [0.9913–1.0391]	1.0023 [0.9855–1.0193]	1.0139 [0.9709–1.0589]
Academic detailer physician or not (physician)				
Step change	− 0.3906 [− 0.9401; 0.1588]	0.9436 [0.8903–1.0001]	0.9551** [0.9231–0.9883]	0.9473 [0.8550–1.0496]
Change in trend	0.0488 [− 0.0975; 0.1951]	0.9966 [0.9747–1.0190]	0.9991 [0.9773–1.0214]	1.0072 [0.9687–1.0473]

*Step change* difference in step change between the intervention and control group immediately after the intervention; *change in trend* difference between the change in trend between the intervention and control group after the intervention; *SO1* average defined daily dose of pain relief medication per patient reimbursed for an analgesic or NSAID per month; *SO2* proportion of patients reimbursed for paracetamol; *SO3* proportion of patient reimbursed for any nonsteroidal anti-inflammatory drug (NSAID); *SO4* proportion of patients reimbursed for both an NSAID and a proton-pump inhibitor among those reimbursed for an NSAID for the first time in 3 months; *β*_*6*_ difference in step change due to the intervention; *β*_*7*_ difference in the change in trend due to the intervention; *p value < 0.05; **p value < 0.01; *underlined* < 0.05 in all four analyses (ITT, ITT-V1, ITT-V2 and PPR)

**Fig. 5:**
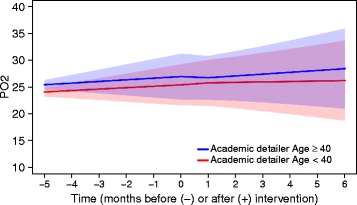
Predicted dose (95% confidence bounds) for PO2 in intervention group over time. PO2, average defined daily dose of paracetamol per patient reimbursed for paracetamol per month

**Fig. 6:**
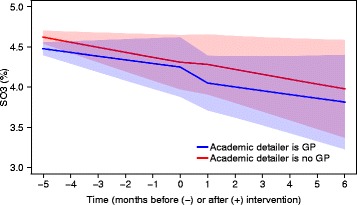
Predicted proportion (95% confidence bounds) for SO3 in intervention group over time. SO3, proportion of patients reimbursed for any nonsteroidal anti-inflammatory drug; GP, general practitioner

The average DPM of paracetamol shifted downward at the time of the intervention when the academic detailer was aged 40 or above (Fig. 5). The odds of being reimbursed for an NSAID shifted downward at the time of the intervention when the academic detailer was a physician (Fig. 6). Note that, although a significant shift was observed at the time of the intervention for both outcomes, the predicted outcomes did not differ between visited practices.

### Sensitivity analyses

When removing the practices for which no date of visit was recorded from the analysis, similar results were obtained for both the primary and secondary outcome analyses and the exploratory analyses (results not shown).

## Discussion

In this study, we assessed the effectiveness of Farmaka’s AD visits on appropriate prescribing of pain relief medication in osteoarthritis. We found a significant impact of the visits conducted by academic detailers on one primary outcome, i.e. the proportion of patients prescribed with a recommended NSAID among those prescribed with any NSAID, confirming the usefulness of the ADS in improving appropriate prescribing of pain relief medication. This result supports the effectiveness of the ADS in general, as demonstrated earlier both in Belgium [6, 7] and abroad [25, 26]. However, an impact of the ADS could not be found for the other outcomes under study, indicating that improvements might be very condition and outcome-specific, which might explain why Borgermans et al. and Goldberg et al. could not demonstrate the effectiveness of the ADS in their countries [8, 27]. Another explanation might be that the advice to change the prescribed drug to a similar safer drug is more easily accepted than the advice to stop prescribing this drug altogether.

Additionally, we found that the profile and age of the AD visitor might impact the effectiveness of the visit. A small, but significant, decrease in the DPM of paracetamol directly after the intervention was observed when the visit was conducted by a visitor aged 40 or above (compared to a visitor aged below 40). Another small, but significant, decrease in the odds of receiving an NSAID directly after the intervention was observed when the visit was conducted by a GP (compared to a visitor who is not a GP). A possible explanation for these traits to positively influence the effectiveness of the AD visit might be that GPs are more inclined to believe a more experienced person, either by training (GP) or by age. Although this was not formally tested, visiting at least one GP in a practice seems to have a comparable impact as visiting all GPs in a practice.

### Strengths and weaknesses of the study

This study used a cluster-randomized controlled trial to evaluate the effectiveness of visits by academic detailers. The cRCT is a well-known and trustworthy design, with its objectiveness increased due to blinding of participating practices. Because all practices (and GPs) had received a visit by an academic detailer in the past, they will not have identified the academic detailer’s visit as an intervention, which ensures that the evidence obtained through this trial is real-world evidence, adding to the trustworthiness of the study results. Another major strength of this study is the size of the group of participating practices, which is quite large and hence adds to the validity and precision of the results found in this study.

A major weakness of the study is that this study focuses on reimbursement data while paracetamol is also available over the counter in Belgium. For this reason, we miss information on paracetamol use. We do however assume that patients with chronic arthritis would need chronic pain relief and hence would buy their paracetamol on prescription, because this is the only way to get a reimbursement for paracetamol. Also note that reimbursement data do not include an indication for the use of pain relief medication. Therefore, we are not absolutely sure that the pain relief medication is used for chronic pain relief in osteoarthritis. To increase the probability that the majority of our patients are osteoarthritis patients, we only considered reimbursement data for patients aged 60 or above. As a result, we assessed the effect of AD visits on use of pain relief medication in general, i.e. in all 60+ patients and not only in those with osteoarthritis.

At the moment of the AD visits, there was also no consensus on the recommended use and safety of paracetamol. In most guidelines, especially at the time of our intervention, paracetamol is suggested as the first-line treatment for chronic osteoarthritis pain (e.g. NICE 2014). However, its effectiveness in pain management of osteoarthritis is currently under debate. A recent systematic review identified (only) seven studies on the use of paracetamol on chronic osteoarthritis pain and found negligible clinical efficacy of continuous dosing regimens of more than 2 weeks, compared with placebo [28]. Similarly, a large meta-analysis showed no or only a very small effect on pain at doses up to 4000 mg/day [29]. Also, the academic detailers themselves referred to the topic as being not up to date [10]. In this respect, it is conceivable that practitioners decide not to follow or not to keep following the recommendation to prescribe paracetamol as a first choice treatment, if their clinical experience and the feedback of their real-world patients are not positive. For this reason, all findings related to KM2 (PO2 and SO2) should be interpreted with great caution. We would expect to find an increased impact of the ADS on topics for which there is more consensus, and future research should focus on verifying the real-world evidence discussed here assessing an AD visit on another topic.

Additionally, this study was not able to cover all Belgian practices. About 24% of practices randomized to the intervention group were not reached because no GP could be visited with this topic. Because we focus our main findings on effects that were significant in all four scenarios, and these 24% of practices that were not visited in the intervention group were only (partly) left out of the analysis in (the ITT-V2 and) the PPR scenario, this has no implications on the findings discussed here. Note that these practices differed from the practices that did receive a visit only in the number of previously received visits, with a higher number of previously received visits rendering higher odds of being visited with this topic. This finding suggests that there might be a benefit to familiarity with either the visitor or the ADS. At the time of the study, Farmaka reached about one in three active GPs in Belgium with their ADS [30]. These practices are not reached, because they did not receive a visit by an academic detailer before. These practices are however similar in baseline characteristics to the practices that were reached, and absence of this study group will most likely not have a major impact on the findings reached here. As Anthierens et al. demonstrated, AD visits which are appreciated by GPs, reaching these unvisited practices, and not necessarily all its GPs, would increase the nationwide impact of Farmaka’s ADS [10]. Meanwhile, half of the Belgian GPs are being visited. Another limitation of the study is that it only reveals short-term effects, as we considered only 6 months after the intervention. This implies that the sustainability of effects more than 6 months after the AD visit could not be assessed.

## Conclusions

The ADS provided by Farmaka was effective in improving the proportion of recommended NSAIDs prescribed by GPs. An impact on overall prescription rates of analgesics and NSAIDs was not detected. The implementation of the ADS could be improved further by selecting academic detailers based on their profile and age.

## Appendix

**Table 6:** Summary of impact of academic detailing visits on primary outcomes

	ITT1	ITT2	PPR
	Step change [95% Wald CI]		
Odds of being reimbursed for an analgesic or NSAID, in intervention compared to control group (PO1)	0.9973 [0.9860; 1.0088]	0.9929 [0.9780; 1.0081]	1.0218 [0.9982; 1.0459]
Average defined daily dose of paracetamol per patient reimbursed for paracetamol per month, in intervention compared to control group (PO2)	− 0.3106 [− 0.8493; 0.2281]	− 0.2442 [− 0.8677; 0.3793]	− 0.2665 [− 0.8491; 0.3161]
Odds of being reimbursed for a recommended NSAID when reimbursed for any NSAID, in intervention compared to control group (PO3)	1.1887* [1.1004; 1.2841]	1.2158* [1.1276; 1.3109]	1.2158* [1.1276; 1.3109]
Odds of being reimbursed for an NSAID and a PPI when reimbursed for an NSAID, in intervention compared to control group (PO4)	1.0194 [0.9419; 1.1032]	1.0188 [0.9336; 1.1117]	1.0050 [0.9534; 1.0593]
	Change in trend [95% Wald CI]		
Odds of being reimbursed for an analgesic or NSAID, in intervention compared to control group (PO1)	0.9882* [0.9790; 0.9975]	0.9896* [0.9806; 0.9987]	0.9990 [0.9945; 1.0035]
Average defined daily dose of paracetamol per patient reimbursed for paracetamol per month, in intervention compared to control group (PO2)	0.0389 [− 0.1446; 0.2224]	0.0937 [− 0.0686; 0.2560]	0.1400 [− 0.0513; 0.3313]
Odds of being reimbursed for a recommended NSAID when reimbursed for any NSAID, in intervention compared to control group (PO3)	0.9902 [0.9775; 1.0030]	0.9894 [0.9776; 1.0013]	0.9882 [0.9753; 1.0012]
Odds of being reimbursed for an NSAID and a PPI when reimbursed for an NSAID, in intervention compared to control group (PO4)	0.9940 [0.9765; 1.0118]	0.9944 [0.9762; 1.0129]	0.9968 [0.9770; 1.0170]

*Step change* difference before and after the intervention; *change in trend* monthly change after the intervention; *CI* confidence interval; *NSAID* nonsteroidal anti-inflammatory drug; *PPI* proton pump inhibitor; *ITT1* adjusted intention to treat analysis after dropping contaminated controls; *ITT2* adjusted intention to treat analysis after dropping contaminated controls and fully contaminated cases; *PPR* per protocol analysis; **p* value < 0.05

**Table 7:** Summary of impact of academic detailing visits on secondary outcomes

	ITT1	ITT2	PPR
	Step change [95% Wald CI]		
Average defined daily dose of analgesic and NSAIDs per patient reimbursed for an analgesic or NSAID per month, in intervention compared to control group (SO1)	0.0640 [− 0.1916; 0.3196]	0.1090 [− 0.1484; 0.3664]	0.2186 [− 0.0707; 0.5079]
Odds of being reimbursed for paracetamol, in intervention compared to control group (SO2)	1.0081 [0.9932;1.0232]	0.9976 [0.9811; 1.0144]	1.0289 [0.9941; 1.0649]
Odds of being reimbursed for an NSAID, in intervention compared to control group (SO3)	1.0078 [0.9943; 1.0214]	1.0055 [0.9918; 1.0194]	1.0269* [1.0033; 1.0510]
Odds of being reimbursed for both an NSAID and a PPI when reimbursed for an NSAID for the first time in 3 months, in intervention compared to control group (SO4)	1.0423 [0.9689; 1.1212]	1.0482 [0.9621; 1.1420]	1.0155 [0.9532; 1.0819]
	Change in trend [95% Wald CI]		
Average defined daily dose of analgesic and NSAIDs per patient reimbursed for an analgesic or NSAID per month, in intervention compared to control group (SO1)	0.0455 [− 0.0183; 0.1094]	0.0316 [− 0.0369; 0.1001]	0.0374 [− 0.0470; 0.1218]
Odds of being reimbursed for paracetamol, in intervention compared to control group (SO2)	0.9867* [0.9766; 0.9968]	0.9889* [0.9785; 0.9994]	0.9960 [0.9871; 1.0049]
Odds of being reimbursed for an NSAID, in intervention compared to control group (SO3)	0.9907 [0.9801; 1.0015]	0.9907 [0.9809; 1.0006]	0.9998 [0.9925; 1.0072]
Odds of being reimbursed for both an NSAID and a PPI when reimbursed for an NSAID for the first time in 3 months, in intervention compared to control group (SO4)	0.9907 [0.9539; 1.0289]	0.9972 [0.9564; 1.0398]	1.0037 [0.9542; 1.0557]

*Step change* difference before and after the intervention; *change in trend* monthly change after the intervention; *CI* confidence interval; *NSAID* nonsteroidal anti-inflammatory drug; *PPI* proton pump inhibitor; *ITT1* adjusted intention to treat analysis after dropping contaminated controls; *ITT2* adjusted intention to treat analysis after dropping contaminated controls and fully contaminated cases; *PPR* per protocol analysis; **p* value < 0.05

## Additional file

Additional file 1: Figure A1. Information package on appropriate use of pain relief medication in patients with osteoarthritis used by the academic detailer (PDF 1229 kb)

## Abbreviations

A02BC: ATC code for proton pump inhibitor
AD: Academic detailing
ADS: Academic detailing service
ATC: Anatomic therapeutic chemical
CME: Continuing medical education
cRCT: Cluster-randomized controlled trial
DDD: Defined daily dose
DPM: Number of defined daily doses per patient per month
GEE: Generalized estimating equations
GI: Gastrointestinal
GP: General practitioner
ITT: Intention to treat
KM: Key message
M01A: ATC code for nonsteroidal anti-inflammatory drugs
M01AE01: ATC code for ibuprofen
M01AE02: ATC code for naproxen
N02: ATC code for general analgesics
N02BE01: ATC code for paracetamol
NSAID: Nonsteroidal anti-inflammatory drug
PO: Primary outcome
PPI: Proton pump inhibitor
PPR: Per protocol
RCT: Randomized controlled trial
SO: Secondary outcome
