# Supplementary material for: The implementation of academic detailing and its effectiveness on appropriate prescribing of pain relief medication: a real-world cluster randomized trial in Belgian general practices
Source: Implement Sci. 2018 Jan 10;13:6. doi: 10.1186/s13012-017-0703-8 (PMC5761112; doi:10.1186/s13012-017-0703-8)
Supplement: Additional file 1: Figure A1. — Information package on appropriate use of pain relief medication in patients with osteoarthritis used by the academic detailer (PDF 1229 kb) [file 13012_2017_703_MOESM1_ESM.pdf]

Aanpak van artrose

FEBRUARI 2013

farmaka

Vzw Farmaka asbl – Onafhankelijk centrum voor geneesmiddeleninformatie

Onafhankelijke artsenbezoekers

Lic. Baitar Abdelbari
Dr. Evens Bart
Apr. Goyen Josée
Apr. D'Hooghe Beatrijs
Dr. Apr. Lobeau Marieke
Lic. Muylle Birgit
Dr. Ir. Van Den Maagdenberg Karijn

Ph. De Ruyck Isabelle
Dr. de Schaetzen Sybille
Dr. Devillers Catherine
Dr. Lacroix Sophie
Biol. Leroy Thérèse
Ph. Nonneman Annick
Ph. Pinckaers Nathalie
Ph. Vanvolsem Clarisse
Dr. Verhaeghen Myriam
Dr. Veys Catherine

WZC-formularium: www.formularium.be
Geneesmiddelenbrief

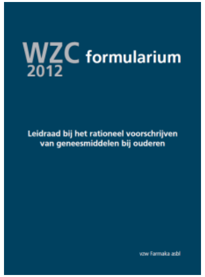

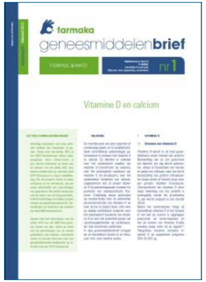

Transparantiefiches BCFI

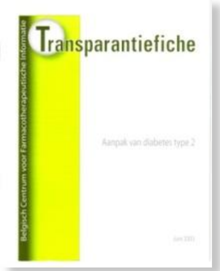

Systematisch literatuuronderzoek
Consensusvergadering RIZIV

farmaka

Inleiding

Keuze van het thema

- Demografisch
- Talrijke therapeutische keuzes
- Anti-inflammatoire middelen: veiligheid

Inhoud

- Artrose van de perifere gewrichten
- Behandeling van pijn, vooral medicamenteus

Doel

- Risico-baten afwegen
- Interactief

EBM

Evidence Based Medicine

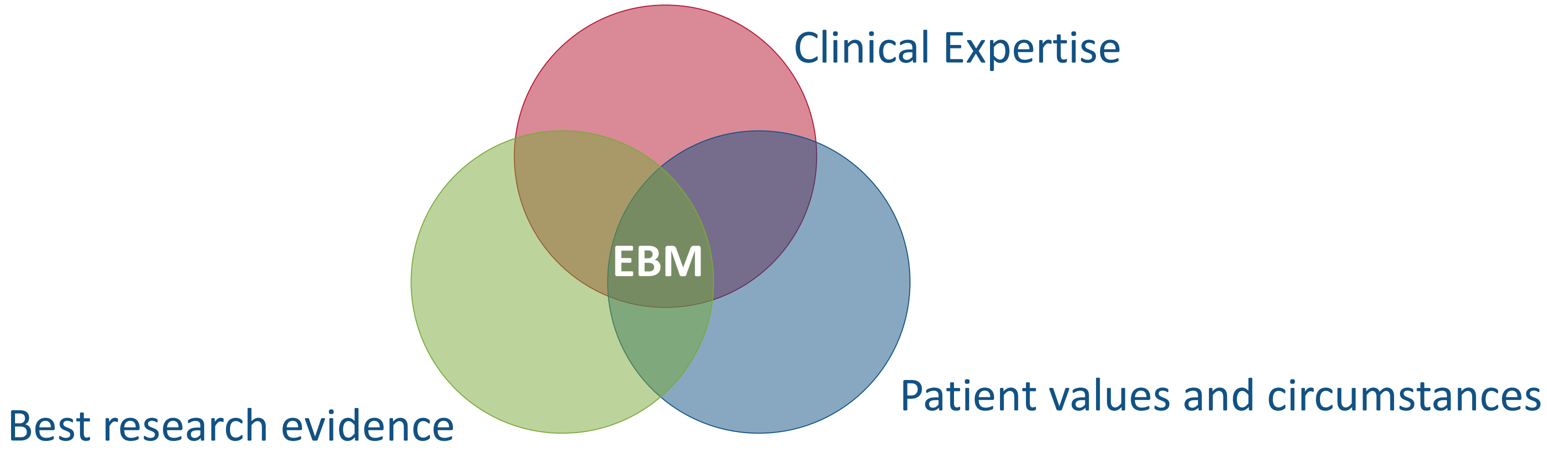

Evidence-based medicine (EBM) requires the integration of the best research evidence with our clinical expertise and our patient's unique values and circumstances

farmaka

Methodologie

Literatuuronderzoek gebaseerd op

- Onafhankelijke geneesmiddelenbulletins van de laatste 5 jaar
- Big five (laatste 5 jaar)
- Clinical Evidence
- Guidelines : EULAR 2003-2005-2007, NHG 2008, CBO 2007, HTA 2006-2008-2011, NICE 2008
- Consensusconferenties 2004 et 2007
- Cochrane database of systematic reviews
- WZC Formularium 2012

Artrose - Inhoudstafel

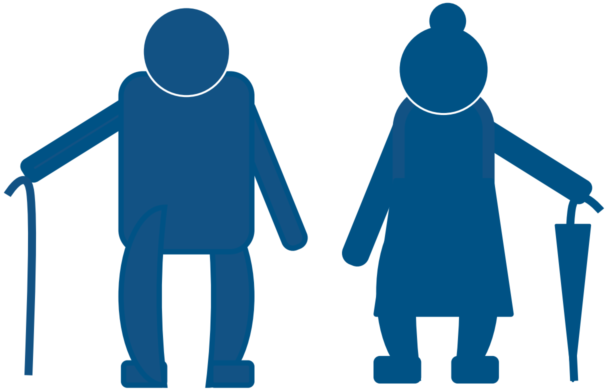

Niet-medicamenteuze interventies

Geneesmiddelen

- Paracetamol
- NSAID per os
- NSAID voor lokaal gebruik
- Infiltratie met glucocorticoïden
- Narcotische analgetica

Annex

Samenvatting

Niet-medicamenteuze interventies

1e stap

Aanbevelingen

- Informatie over natuurlijk verloop
  - Niet noodzakelijk negatief - afhankelijk van locatie van het gewricht

- Levensstijl
  - Regelmatige fysieke activiteit/specifieke oefeningen — Knie; 1MA, N=13
  - ↘ gewicht — Knie; 1MA, N=5, n=454; 1RCT, n=316
  - Wandelstok, aangepaste schoenen, brace… — Knie, heup, hand; 1MA, N=5, n=444, 12RCT
  - Eventueel warmte of koude applicatie — Koude: knie; MA, N=3, n=179

farmaka

NICE 2008, Belo 2008, Vignon 2006, Bijlsma 2011, CKS 2008, Roddy 2005, Christensen 2007, Rejeski 2002, Brouwer 2005

Geneesmiddelen

Paracetamol = 1e keuze

Paracetamol	• 1e keuze – goede risico/baten verhouding
NSAID per os	• Indien onvoldoende respons op paracetamol<br>• Keuze : ibuprofen of naproxen
NSAID voor lokaal gebruik	• Plaats bij artrose van de kleine gewrichten
Glucocorticoïd infiltratie	• Plaats bij artrose van de knie
Zwakke of matig krachtige narcotische analgetica	• Zeer beperkte plaats<br>• Keuze: paracetamol + codeine

farmaka

Paracetamol

1e keuze – bewezen werkzaamheid – goed veiligheidsprofiel

TERUGBETALING

- **Werkzaamheid**
  - Verbetering van de pijn

WERKZAAMHEID

1 SR (N=7 n=2491)

2 RCT (n=325, n=483)

- **Maximale dosis**

	Zonder risicofactoren	Met risicofactoren
Acuut	4g/d	3g/d
Chronisch (>1maand)	2,5g/d	2g/d

RISICOFACTOREN

- **Veiligheid**
  - Hepatotoxiciteit
  - INR

DETAILS VEILIGHEID

farmaka

Towheed 2006, Verduijn 2004, BCFI 2011

NSAID per os

Indien onvoldoende respons op paracetamol

WERKZAAMHEID
VEILIGHEID

- **Versus paracetamol ->Risico/baten verhouding minder gunstig**
  - Werkzamer, maar beperkte meerwaarde, studies korte termijn

Risico gastro-intestinaal - cardiovasculair - renaal
Geef zo kort mogelijk – Doseer zo laag mogelijk

- **NSAID's onderling**
  - **Werkzaamheid:** geen verschil tussen NSAID's

    → 1e keuze gebaseerd op veiligheidsprofiel, farmacovigilantie, prijs

Ibuprofen

farmaka

Towheed 2006, Chou 2011, Henry 1996, Gonzalez 2010, WZC 2012

NSAID – Gastro-intestinaal risico

Verhoogd risico met alle NSAID's

- Ibuprofen lage dosis: laagste risico van klassieke NSAID's
- COX-2 'selectieve' NSAID's vs klassieke NSAID's
  - Minder gastro-duodenale ulcera en ulcuscomplicaties op korte termijn
  - Geen verschil indien klassieke NSAID + PPI
  - CV risico!

→ 1e keuze : ibuprofen – Aanbeveling: max 1200mg/d

+ PPI indien gastro-intestinale risicofactor

ENKELE CIJFERS

VERGELIJKING KLASSIEKE

COX-2

GI RISICOFACTOREN TERUGBETALING PPI

Gonzalez 2010, Henry 1996, Chou 2011, CKS 2010

NSAID – Cardiovasculair risico

Kan voor geen enkel NSAID worden uitgesloten

- Ibuprofen lage dosis (≤ 1200mg) en naproxen: gegevens geruststellend
- Diclofenac : hoogste risico van de klassieke NSAID's ←EMA-evaluatie
- COX-2 'selectieve' NSAID's: risico hoger dan klassieke NSAID's

DETAILS COX 2

Ideaal geen NSAID bij cardiovasculaire pathologie/risicofactor

CV RISICOFACTOREN

→ Indien nodig: ibuprofen (max 1200mg/d) of naproxen (max 1000mg/d)

farmaka

Altman 2007, Chen 2008, Garcia Rodrigues 2008, Fosbol 2009, Chou 2006, Hippisley Cox 2005, Trelle 2011, EMA 2012

NSAID – Renaal risico en andere

RENALE RISICOFACTOREN

ALLE RISICO'S

- **Renaal**
  - Nierinsufficiëntie
  - Vochtretentie → verergeren hartfalen, ↗ bloeddruk
- **↗ kaliëmie**
  - Kaliumsupplementen, kaliumsparend diureticum, ACE-I, sartaan, aliskiren, heparine

Ideaal geen NSAID indien
- (risicofactor) nierinsufficiëntie
- (behandeling van) hartfalen
- antihypertensieve behandeling

farmaka

BCFI 2013, Prescrire 2011, NICE 2008

NSAID voor lokaal gebruik

Plaats bij artrose van de kleine gewrichten

- **Werkzaamheid**
  - Werkzaam vs placebo
  - Vs NSAID per os: per os > topisch op korte termijn (1w),
    geen verschil middellange termijn (2-4w)
- **Veiligheid**
  - Plasmaconcentraties lager dan per os, maar niet zero
  - Lokale huidreacties waaronder fotosensibiliteit
- **Richtlijnen**
  - Hand: als eerste stap, of als alternatief voor paracetamol of NSAID per os
- **Praktische overwegingen**
  - 3 - 4x/d aanbrengen, prijs

NICE 2008, Altman 2009, Lin 2004 , Zacher 2001 , Belo 2008,, EULAR 2003, EULAR 2007, Zhang 2007

Infiltraties met glucocorticoïden

Knie – indien onvoldoende respons op analgetica per os

- **Werkzaamheid**
  - Knie: werkzaamheid aangetoond maar beperkt in de tijd
  - Heup – hand : beperkte onderbouwing
- **Veiligheid**
  - Lokale en systemische ongewenste effecten
- **Aanbeveling**
  - Max 3x/jaar/gewricht

Bellamy 2006, NICE 2008, CKS 2010, Belo 2008

Zwakke of matig krachtige narcotische analgetica

Zeer beperkte plaats

Indien R/: voorkeur paracetamol + codeine

DETAILS WERKZAAMHEID

DETAILS VEILIGHEID

- **Werkzaamheid**
  - Zwakke onderbouwing
  - → Geen gegevens die superioriteit aan paracetamol of NSAID suggereren
- **Veiligheid**
  - Meer ongewenste effecten en stoppen omwille van ongewenste effecten (vs placebo, vs paracetamol, vs NSAID)
  - Meer risico van interacties met tramadol (farmacologisch)
  - Risico van afhankelijkheid

Nüesch 2009, Cepeda 2006, Kjaersgaard 1990, Boureau 1990, Bianchi 2003, Pavelka 1998, Dalgin 1997

Samenvatting

Niet medicamenteuze aanpak = 1e stap

Paracetamol = 1e keuze indien medicatie

NSAID per os

Evalueer gastro-intestinaal, cardiovasculair, renaal risico van de patiënt met alle NSAID's

- 1e keuze: ibuprofen max 1200mg
- Indien cardiovasculair risico

mogelijk alternatief: naproxen max 1000mg

+PPI indien gastro-intestinaal risico

NSAID voor lokaal gebruik: plaats bij artrose van de kleine gewrichten

Glucocorticoïd infiltratie: plaats bij artrose van de knie

Zwakke of matig krachtige narcotische analgetica: zeer beperkte plaats - keuze: paracetamol + codeine

Annex

Annex – Inhoudstafel

Beperkingen studies	Paracetamol	NSAID per os	Klassieke NSAID’s
Evolutie - Diagnose	Werkzaamheid	Werkzaamheid	GI risico – vergelijking
NSAID - Lijst	Veiligheid	GI risico - cijfers	Cox-2 ‘selectieve’
	Risico hepatotoxiciteit	GI risicofact/terugbet. PPI	GI risico
	Terugbetaling	CV risicofactoren	CV risico
		Risicofactoren nier	
		Veiligheid	NSAID + ASA

Narcotische analgetica	Andere	
Werkzaamheid	Glucosamine - Chondroïtine	Hyaluronzuur
Veiligheid		Nabumeton

Beperkingen van de studies

De meeste studies

- Knie
- Korte duur (<3 maand)
- Exclusie
  - Zeer oude patiënten
  - Ernstige artrose
  - Ernstige comorbiditeit (o.a. cardiovasculaire voor COX-2 ‘selectieve’ NSAID's)
- Dagelijkse therapeutische dosis (in plaats van gebruik bij opstoten zoals in de praktijk)

Artrose – Evolutie & Diagnose

- **Natuurlijk verloop**
  - Afhankelijk van locatie van het gewricht
  - Opstoten afgewisseld met periodes met minder klachten
- **Klinische diagnose** (>50 jaar)
  - ↗ pijn bij belasting van het gewricht
  - Ochtendstijfheid < 30 minuten
  - Benige verbreding van het gewricht
  - Afwezigheid van verhoogde temperatuur en afwezigheid van belangrijke zwelling
  - Crepitaties bij bewegingsonderzoek
- **Radiologie** eventueel nuttig bij diagnostische twijfel

NICE 2008, CBO 2007

Paracetamol - Terugbetaling

	Voorwaarden	Aanvraagformulier	Max. duur bij 1ste aanvraag	Max. duur per verlenging
**Magistraal**	Chronische pijn<br>Evt. in associatie[1]	Diagnose ziekte	12 maanden	12 maanden
**'Chr' BCFI**	Chronische pijn[2]<br>> 3 maanden<br>Evt. in associatie met codeine	Kennisgeving "chronische pijn"	12 maanden	12 maanden
**b ▶! BCFI<br>Bf hoofdstuk IV**	Chronische pijn[2]<br>> 6 maanden<br>Max. 3 g per dag	Specifiek aanvraagformulier<br>§ 5460000	12 maanden	60 maanden

1 Associatie kan met acetylsalicylzuur, coffeine en codeine

2 Kankerpijn; chronische arthritis/artrosepijn; neuropatische pijn van centrale of perifere oorsprong (met inbegrip van multiple sclerose); perifere vasculaire pijn; postchirurgische pijn (met inbegrip van fantoompijn); fibromyalgie

Paracetamol - Terugbetaling

[paracetamol]

compr. (deelb.)			
10 x 500mg		Chr	€ 1,50
100 x 500mg	Rx	b!	€ 8,03
10 x 1g		Chr	€ 2,20
50 x 1g	Rx	b!	€ 8,03
90 x 1g	Rx	b!	€ 10,52
bruiscompr.			
32 x 500mg	Rx	Chr	€ 4,75
bruiscompr. (deelb.)			
20 x 1g	Rx	Chr	€ 5,50
40 x 1g	Rx	Chr	€ 9,50
60 x 1g	Rx	b!	€ 8,77
poeder (zakjes)			
40 x 1g	Rx	Chr	€ 9,50
60 x 1g	Rx	b!	€ 8,77

(compr. (deelb.) 50 x 1 g en 90 x 1 g, buiscompr., buiscompr. (deelb.) en poeder (zakjes) ook op schriftelijke aanvraag van de patiënt)

Paracetamol - Werkzaamheid

Cochrane Towheed 2006						
Design	Populatie	Interventie	N/n	Duur	Eindpunten	Resultaten
SR van RCTs	Volwassene met primaire of secundaire artrose. Knie- en heupartrose. Gemiddelde leeftijd 62 jaar.	Paracetamol (4 x 650-1000mg/d) vs placebo	7/2491	Gemiddeld 6w		
		4 x 1000mg /d	5/1835		Pijn	**SMD= -0.13 (95%BI -0.22 tot -0.04)**
			6/2385		Aantal patiënten met ongewenste effecten	318/1239 vs 290/1146 RR=1.02 (95%BI 0.89 tot 1.17)
Opmerkingen: Studies evalueerden effect paracetamol aan de hand van verschillende meetinstrumenten op pijn, functioneren, globaal welbevinden: WOMAC, MDHAQ VAS, Lequesne, …						

Effect size	
< 0,2 SD	zwak
0,5 SD	matig
> 0,8 SD	sterk

farmaka

Paracetamol – Risicofactoren hepatotoxiciteit

Dosering: Max 3g/d acuut– 2g/d chronisch

Chronisch alcoholgebruik	> 4 eenheden/d
CYP2E1-enzyminducerende middelen	Isoniazide, rifampicine, carbamazepine, phenytoïne, fenobarbital
CYP3A4 -enzyminducerende middelen	Sint-Janskruid
Deficiënte voeding	(Eiwitarm) dieet / slechte voedingstoestand / vasten<br>Gewicht <50 kg
Gecombineerd gebruik van pijnstillers	Vb. in combinatiepreparaten of met NSAID's of opioïden
Hogere leeftijd	(↘ metabolisatiesnelheid)
Leverfunctiestoornissen	CI paracetamol: ernstige leverinsufficiëntie
Nierfunctiestoornissen	

Risico is cumulatief!

Verduijn 2004, BCFI 2011

Paracetamol – Veiligheid

- **Hepatotoxiciteit**
  - Problemen te verwachten ≥10g/d
  - Chronisch gebruik: meeste gevallen van toxiciteit bij dosis >4g/d
  - Opgelet bij risicofactoren van hepatotoxiciteit
    - Toxiciteit mogelijk bij lagere doses
    - Cumulatief effect

- **INR ontregeling**
  - Bij chronisch gebruik >2g/d

- Analgetica-nefropathie bij chronisch gebruik ?
  - In het verleden: overgebruik combinatiepreparaten
  - Monotherapie : weinig aanwijzingen, geen causaal verband aangetoond

Michels 2010, Rahme 2002, Chou 2011, BCFI 2013

NSAID – Werkzaamheid

Cochrane Towheed 2006						
Design	Populatie	Interventie	N	Duur	Eindpunten	Resultaten
SR van RCTs	Volwassene met primaire of secundaire artrose. Knie- en heupartrose. Gemiddelde leeftijd 62 jaar.	NSAID's (ibuprofen, diclofenac, celecoxib, naproxen, rofecoxib) vs paracetamol (4 x 650-1000mg)	10	Gemiddeld 6 w		
			8/2358		Pijn	**SMD: -0.25 (95%BI -0.33 tot -0.17)**
			2/832		Functioneren (WOMAC)	**SMD: -0.25 (95%BI -0.40 tot -0.11)** Geen verschil op HAQ of Lequesne index.
		NSAID (ibu. of napr.) vs paracetamol	5/891		Patiënten met ongewenste GI effecten	**19% vs 13%** **RR=1.47 (95%BI 1.08 tot 2.00)** **NNH=12 (95%BI 6 tot 66)**
		NSAID (ibu., diclo. of napro.) vs paracetamol	5/640		Uitval door ongewenste GI effecten	**8% vs 4%** **RR=2.00 (95%BI 1.05 tot 3.81),**

Opmerkingen: Studies evalueerden effect paracetamol en NSAID's aan de hand van verschillende meetinstrumenten op pijn, functioneren, globaal welbevinden: WOMAC, MDHAQ VAS, Lequesne
Veiligheid: moeilijk te evalueren op korte periode

Effect size	
< 0,2 SD	zwak
0,5 SD	matig
> 0,8 SD	sterk

farmaka

Klassieke NSAID's – gastro-intestinaal risico

Lage dosis ibuprofen: laagste risico

Geschat relatief risico op gastrointestinale ongewenste effecten met klassieke NSAID’s (vergeleken met ibuprofen) – observationele studies		
Ibuprofen (lage dosis*)	1 (referentie)	
Acetylsalicylzuur	1.6	1.3-2.0 (95%BI)
Diclofenac	1.8	1.4-2.3
Naproxen	2.2	1.7-2.9
Indometacine	2.4	1.9-3.1
Piroxicam	3.8	2.7-5.2

*as used in clinical practice in seven countries. The differences seemed to be attributable to the fairly low dose of ibuprofen employed in clinical practice. It should not be assumed that the apparent advantage of ibuprofen persists when doses are increased beyond 1600 mg daily. The evidence reviewed indicates that it does not.

farmaka

Henry 1996

NSAID – Gastro-intestinaal risico

**Ernstige gastro-intestinale bloeding met NSAID**	Jaarlijks risico : 1/110 bij > 75 jaar	
**Klassieke NSAID's vs geen gebruik**		
Hoge GI bloeding of perforatie	RR = 4.50 (95%BI 3.82 tot 5.31)	SR obs.
Risico dosisafhankelijk	RR hoge dosis= 5.30 (95%BI 4.73 tot 5.94)<br>RR lage dosis=2.88 (95%BI 2.54 tot 3.27)	SR obs.<br>SR obs.
Risico lager bij langere halfwaardetijd	RR vertraagde vrijstelling= 5.87 (95%BI 4.74 tot 7.26)<br>RR lange $t_{1/2}$ (≥12u) = 5,62 (95%BI 4.76 tot 6.65)<br>RR korte $t_{1/2}$ (<12u) = 3,21 (95%BI 2.83 tot 3.63)	SR obs.<br>SR obs.
**Celecoxib vs klassieke NSAID's**		
Ulcuscomplicaties	RR = 0.23 (95%BI 0.07 tot 0.76)	SR RCT N=3

farmaka

Blower 1997, Gonzalez 2010, Chou 2011

COX-2 'selectieve' NSAID's - Gastro-intestinaal risico

Versus klassieke NSAID's

- ↘ gastro-duodenale ulcera met COX-2 'selectieve'
- ↘ ulcuscomplicaties met celecoxib maar niet op lange termijn (12 maand)
- Ulcuscomplicaties etoricoxib: niet statistisch significant verschillend (18m)

Versus klassieke NSAID's + PPI

- geen statistisch significant verschil of klinisch niet relevant

Aanwijzingen uit studies dat

het potentieel voordeel van COX-2 'selectieve' zou verdwijnen bij concomittant gebruik van lage dosis ASA

DETAILS ASA

Chou 2011, CBO 2007

NSAID – Gastro-intestinale risicofactoren

- Hogere leeftijd (terugbetalingscriterium >65j)
- Ernstige comborbiditeit (cardiaal, diabetes,..)
- Voorgeschiedenis van maagulcera
- Voorgeschiedenis van ulcuscomplicaties (bloeding, perforatie)
- Gelijktijdig gebruik van orale corticosteroïden
- Gelijktijdig gebruik van ASA
- Gelijktijdig gebruik van orale anticoagulantia
- Gelijktijdig gebruik van anti-aggregantia
- Dyspeptische klachten bij NSAID gebruik
- Hoge dosis NSAID
- Gebruik meerdere NSAID's tegelijk
- Helicobacter Pylori
- Gelijktijdig gebruik van SSRI's
- Invaliderende reumatoïde artritis

+ PPI

Esomeprazol 20mg/d
Lansoprazol 30mg/d
Omeprazol 20mg/d
Pantoprazol 20mg/d

Terugbetaald cat B, ter preventie bij risicopatiënten, tijdens de inname van een NSAID
A posteriori controle

farmaka

NICE 2008, RIZIV 2010

NSAID – cardiovasculaire risicofactoren

Ischemisch vaatlijden (hart, centraal, perifeer)

Cardiovasculaire risicofactoren, waaronder arteriële hypertensie

→ Risico (verder) ischemisch vaatlijden

Hartfalen, stenose nierarteries

→ Risico verergeren hartfalen en nierinsufficiëntie

Behandeling met antiaggregans of anticoagulans

→ Risico bloeding, risico 'aspirineresistentie'

Prescrire 2011

COX-2 ‘selectieve’ NSAID's – Cardiovasculair risico

- **Rofecoxib**: ↗ CV events (waaronder myocardinfarct) → teruggetrokken van de markt
- **Etoricoxib**
  - ↗ cardiovasculaire events (RR=2,72 95%BI 1,18 tot 6,27) vs naproxen
  - ↗ cardiovasculair risico → FDA weigerde goedkeuring
- **Celecoxib**
  - ↗ myocardinfart (RR= 1,77 95%BI 1,00 – 3,11) vs klassieke NSAID's
  - 6 studies vroegtijdig afgebroken owv verhoogd risico CV events

**Absolute contra-indicaties**	Ischemisch hartlijden, antecedenten van CVA<br>Perifeer vaatlijden<br>Matig hartfalen<br>Etoricoxib : ook niet-gecontroleerde hypertensie

farmaka

Avorn 2007, Chen 2008, Chevalier 2008, BCFI 2013

NSAID – Renale risicofactoren

Gedaalde nierfunctie, bestaande nierinsufficiëntie	Hypovolemie (deshydratatie, diureticum)<br>Geneesmiddelen die de nierfunctie wijzigen (ACE-I, sartanen, aliskiren,…)
Nefrotisch syndroom	
Hartfalen	Glomerulaire filtratie hangt af van het vasodilaterend effect van prostaglandines<br>Vochtretentie kan hartfalen verergeren
Levercirrose met ascites	

farmaka

Prescrire 2011, BCFI 2013

NSAID – Veiligheid

Ongewenste effecten	Risicofactoren en/of geneesmiddeleninteracties
Gastro-duodenaal ulcus	• Oudere patiënt, belangrijke comorbiditeit, antecedent peptisch ulcus +/- bloeding of perforatie<br>• ASA, bisfosfonaat, cholinesterase-inhibitor, baclofen, corticosteroïd
Gastro-intestinale bloeding	• ASA, corticosteroïden, SSRI, venlafaxine, duloxetine, spironolactone, L-Dopa<br>• Anticoagulantia
↗ INR en antiaggregantie	• Anticoagulantia en antiaggregantia
Arteriële trombose	• Ischemisch vaatlijden (cardiaal, centraal, perifeer)
Nier – ↗ nierinsufficiëntie	• Hypovolemie, hartfalen, levercirrose met ascites, nefrotisch syndroom, arteria renalis stenose<br>• Functionele nierinsufficiëntie: diureticum, ACE-I, sartaan, aliskiren<br>• Organische nierinsufficiëntie: quinolone, lithium, fibraat, exenatide, liraglutide,…
Nier – Vocht-retentie – ↗ NI	• Behandeling van hartfalen
Nier – Vocht-retentie – ↗ hartfalen	• Behandeling van hypertensie
Nier – ↘ renale eliminatie	• Metformine (lactaatacidose), digoxine, lithium, hypoglykemiërende sulfamiden, metotrexaat, …
↗ kaliëmie	• K^+, kaliumsparend diureticum, ACE-I, sartaan, aliskiren, heparine, drospirenon

CI NSAID: gastroduodenaal ulcus

CI NSAID : ernstig hartfalen
CI COX-2 'selectieve': matig hartfalen
CI etoricoxib : niet gecontroleerde hypertensie

Indien behandeling met anticoagulans, (risicofactor) nierinsufficiëntie, antihypertensiva, behandeling van hartfalen: bij voorkeur geen NSAID

Prescrire 2011

farmaka

NSAID - Veiligheid

Ongewenste effecten	Risicofactoren en/of geneesmiddeleninteracties
Neuropsy: hoofdpijn, vertigo, verwardheid	• Frequenter met arylazijnzuurderivaten en indoolderivaten
Hypersensibiliteit : angio-oedeem, bronchospasme, huiderupties	CI NSAID: ATCD astma of urticaria onder NSAID of ASA
Huid : erupties, Stevens-Johnson en Lyell syndroom, fotosensibilisatie	• Ook indien per os, vooral met ibuprofen, naproxen, diclofenac, indometacine, ketoprofen<br>• Cycline, quinolone, sulfamide, antimycotica, antiviraal, tuberculostatica, antimalariamiddelen, cardiovasculaire geneesmiddelen (amiodaron, diureticum, ACE-I,…), psychotropica (Sint-Janskruid, fluoxetine, venlafaxine, carbamazepine, lamotrigine), geneesmiddelen bij inflammatoire darmziekten,…
Verergering van infecties	
Hematologisch : ↘ RBC, WBC, BP, agranulocytose, eosinofilie	
↘ fertiliteit ♀<br>Foetotoxiciteit	• Fertiliteit: in geval van chronisch gebruik (reversiebel)
Hepatotoxiciteit en pancreatitis (zeldz)	• Hepatotoxiciteit: vooral diclofenac

ASA + NSAID

- **↗ risico gastro-duodenaal ulcus**
- **↗ risico bloeding, vooral gastro-intestinaal**

- **ASA zou het gastro-intestinale voordeel van COX-2 ‘selectieve’ NSAID's verminderen** (of doen verdwijnen) (versus klassieke NSAID's – 1 RCT)

- **NSAID's zouden het CV beschermend effect van ASA verminderen** (of doen verdwijnen)

  → Aanbeveling:

  Neem immediate release ASA minimum 30 min voor NSAID

farmaka

Prescrire 2011, Silverstein 2000, CBO 2007, SSPF 2007, BCFI 2009

Zwakke tot matig krachtige **narcotische analgetica - Details**

- **Paracetamol vs paracetamol + codeine**
  - 1 RCT (paracetamol 3x1g/d vs paracetamol 3x1g/d+ codeine 3x60mg/d)
    - Combinatie werkzamer na 1 w, geen verschil na 4 w — n=158
    - Meer gastro-intestinale ongewenste effecten na 4w
  - 1 RCT (paracetamol 1g vs paracetamol 400mg+codeine 25mg) — n=234
    - Geen verschil in werkzaamheid en veiligheid
- **Tramadol** 3x50mg/d **vs paracetamol** 3x500mg/d — n=20
  - Paracetamol werkzamer en minder ongewenste effecten (nausea, braken)
- **Tramadol vs 'klasieke' NSAID's**
  - Tramadol 3x50-100mg/d vs diclofenac 3x25-50mg/d : NS — n=120
  - Tramadol 200-400mg/d vs ibuprofen 1200-2400mg/d : NS — n=onbekend
- **Tilidine**
  - geen studies, belangrijke ongewenste effecten

farmaka

Kjaersgaard 1990, Boureau 1990, Bianchi 2003, Pavelka 1998, Dalgin 1997

Narcotische analgetica - Veiligheid

Ongewenste effecten		Risicofactoren en/of geneesmiddeleninteracties
Gastro-intestinaal waaronder constipatie		• Cholinerg: loperamide, dextromethorfan, anti-emetica, calciumkanaalblokkers,…<br>• Anticholinerg: antiparkinson, antispasmodica bij urinaire incontinetie, bronchodilatator, TCA, sedatieve antihistaminica, neuroleptica, memantine,…<br>• Verandering van de intestinale innervatie : amiodaron, statine, metformine, chronisch alcoholgebruik,…<br>• deshydratatie<br>• Hypokaliemie, diureticum, corticosteroïd, ß2-agonist, theofylline, insuline,…
Neuropsy, waaronder sedatie		• Sedativa
Mictieproblemen		
CV, o.a. ↗↘ hartritme, orthostatische hypotensie		
Afhankelijkheid - Derving		
Tramadol	Serotoninerg	• Serotoninergica: antidepressiva (amitriptyline, SSRI, venlafaxine, duloxetine, mianserine, mirtazapine, Sint-Janskruid, bupropion,…), dextromethorfan, lithium, antimigrainemiddelen,…
	Convulsies	• ↘ convulsiedrempel: psychotrope (neuroleptica, lithium, cholinesterase-inhibitoren, anticholinergica, bupropion, varenicline, antimigrainemiddelen,…), quinolone, aliskiren, indometacine, corticosteroïde
	Hypoglykemie	• Hogere leeftijd, nierinsufficiëntie, diabetes

Prescrire 2011

Glucosamine - Chondroïtine

Niet aanbevolen

- **Werkzaamheid**
  - ↘ pijn : statistisch significant maar klinisch niet relevant
  - ↘ gewrichtsspleetvernauwing: niet significant
- **Veiligheid - Glucosamine**
  - Gastro-intestinale last, hoofdpijn, moeheid, allergische reacties
  - Interacties vit K antagonisten (↗ INR)

Chou 2011, Clegg 2006

Glucosamine

- Effect op pijn statistisch significant maar klinisch niet relevant >2 SR
- Er is geen SS effect in studies die niet gesponsord zijn 2 MA
- Er is geen SS effect in studies met adequate allocation concealment 2 MA
- Er is een SS effect bij studies die glucosamine Rottapharm® evalueren, en niet bij andere 1 MA
  - Samenstelling van het product?
  - Methodologie: meetmethode, verschil in o.a. outcome measures?
  - Methodologische kwaliteit vd studies (oa. bias vb door sponsoring)
  - Methodologische kwaliteit van de meta-analyse (heterogeniteit)

farmaka

Chou 2011, Vlad 2007, Towheed 2005, CKS 2008, NICE 2008

Glucosamine Rotta vs placebo

Analysis 4.1. Comparison 4 Glucosamine versus placebo (Rotta preparation), Outcome 1 Pain.

Review: Glucosamine therapy for treating osteoarthritis

Comparison: 4 Glucosamine versus placebo (Rotta preparation)

Outcome: 1 Pain

toedieningswijze, studieduur en studiegrootte verschillend

Study or subgroup		Glucosamine N	Glucosamine Mean(SD)	Placebo N	Placebo Mean(SD)	Std. Mean Difference IV,Random,95% CI	Weight	Std. Mean Difference IV,Random,95% CI
Crolle 1980	IM; 3w	15	0.21 (0.43)	15	1.13 (0.89)		11.5 %	-1.28 [-2.08, -0.49]
D'ambrosio 1981	IM/PO; 1w/2w	15	0.33 (0.12)	15	1.2 (0.19)		6.5 %	-5.33 [-6.94, -3.71]
Drovanti 1980	PO; 4w	40	0.95 (0.82)	40	1.88 (0.44)		13.6 %	-1.40 [-1.89, -0.91]
Herrero-Beaumont 2007	PO, 24w	106	5.1 (3.03)	104	6 (3)		14.8 %	-0.30 [-0.57, -0.03]
Pavelka 2002	PO, 3y	101	4.61 (3.45)	101	5.03 (3.13)		14.7 %	-0.13 [-0.40, 0.15]
Pujalte 1980	PO, 8w	10	1.25 (0.25)	10	2.36 (0.79)		9.6 %	-1.81 [-2.89, -0.74]
Reginster 2001	PO, 3y	106	156.1 (101.9)	106	164.2 (104.5)		14.8 %	-0.08 [-0.35, 0.19]
Rovati 1997	PO, 12w	79	24.3 (19.3)	77	50 (22)		14.4 %	-1.24 [-1.58, -0.89]
**Total (95% CI)**		**472**		**468**			**100.0 %**	**-1.11 [-1.66, -0.57]**

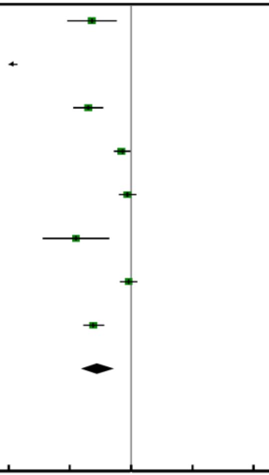

Heterogeneity: $Tau^2 = 0.50$; $Chi^2 = 92.89$, df = 7 ($P<0.00001$); $I^2 = 92\%$

Test for overall effect: Z = 4.01 (P = 0.000061)

Belangrijke heterogeniteit

Towheed 2005

Hyaluronzuur

Niet aanbevolen

- **Werkzaamheid**
  - Zwak effect of geen effect in de studies van goede kwaliteit
  - Belangrijk placebo-effect
  - Heterogene studies (preparaten, injectieschema's)

- **Praktische overwegingen**
  - 3 tot 5 injecties, wekelijkse intervallen
  - Kostprijs : 32,61€ / injectie

farmaka

Divine 2007, Arrich 2005, BCFI 2005

Nabumeton

Geen meerwaarde – niet aanbevolen

- In vitro selectiever COX-2 ? klinisch belang in vivo niet aangetoond
- **Werkzaamheid:** geen verschil vs andere NSAID's
- **Veiligheid:**
  - Minder gastro-intestinale ongewenste effecten?
    - Niveau van bewijskracht onvoldoende
    - Niet dosisafhankelijk? Niveau van bewijskracht onvoldoende
  - Minder risico bij chronisch gebruik?
    - Geen bewijs. Geen verschil vs ibuprofen. Chronisch gebruik afgeraden voor alle NSAID's!
  - Voordeel indien overgevoeligheid NSAID's?
    - Onvoldoende bewijs

Chou 2011, Prescrire 1999, Fleischmann 1992, Morgan 1993, Carle 1992, Macdonald 1997, Prieto 2007

NSAID - Indeling

**Arylazijnzuurderivaten**	Aceclofenac	Air-Tal®, Biofenac®, generieken
	Diclofenac	Cataflam®, Motifene®, Polyflam®, Voltaren®, generieken
	Ketorolac	*Taradyl®*
**Arylpropionzuurderivaten**	Dexketoprofen	Enantyum®, Ketesse®
	Ibuprofen	Brufen®, Dolofin®, Epsilon®, Ibumed®, Malafene®, Nurofen®, Optalidon®, Perdofemina®, Perdophen®, Spidifen® , generieken
	Ketoprofen	Bi-Rofenid®, Rofenid®
	Naproxen	Aleve®, Apranax®, Naproflam®, Naprosyne®, generieken
	Oxaprosine	Duraprox®
**Indoolderivaten**	Indometacine	Dolcidium®
	Proglumetacine	Tolindol®
**Oxicams**	Meloxicam	Mobic®, generieken
	Piroxicam	Brexine®, Feldene®, Polydene®, Solicam®, generieken
	Tenoxicam	Tilcotil®
**COX-2-selectieve**	Celecoxib	Celebrex®
	Etoricoxib	Arcoxia® , Ranacox®
**Nabumeton**		Gambaran®

farmaka

Referenties

- Altman EM, B. J. (2007). Use of Nonsteroidal Antiinflammatory drugs: An update for clinicians: A Scientific Statement from the American Heart Association. *Circulation* , 115:1634-1642.
- Altman R. (2009). Diclofenac sodium gel in patients with primary hand osteoarthritis:a randomized, double-blind, placebo-controlled trial. *J Rheumatol* , 36:1991-9.
- Arrich J, P. F. (2005). Intra-articular hyaluronic acid for the treatment of osteoarthritis of the knee. Systemic review and meta-analysis. *Can Med Ass J* , 172: 1039-43.
- Avorn J. (2007). Keeping science on top in drug evaluation. *N Engl J Med* , 357: 633-5.
- Bellamy N, (2006). Intraarticular corticosteroid for treatment of osteoarthritis of the knee. . *Cochrane Database of Systematic Reviews* , Issue 2. Art. No.: CD005328. DOI: 10.1002/14651858.CD005328.pub2.
- Belo JN, B.-Z. S. (2008). NHG-Standaard Niet-traumatische knieproblemen bij volwassenen (Eerste herziening). *Huisarts Wet* , 51(5):229-40.
- Bianchi M, B. M. (2003). Effects of tramadol on synovial fluid synovial fluid concentrations of substance P and interleukin-6 in patients with knee osteoarthritis: comparison with paracetamol. *International Immunopharmacology* , 3 (13-14): 1901-8.
- Bijlsma J, B. F. (2011). Osteoarthritis: an update with relevance for clinical practice. *Lancet* , 377:2115-26.

farmaka

- Blower A, B. A. (1997). Emergency admissions for upper gastrointestinal disease and their relation to NSAID use. *Aliment Pharm Ther* , 11(2):283-91.
- Boureau F, D. G. (1990). Comparative study of the efficacy and tolerance of 2 dosages of the paracetamol 400mg codeïne 25mg assocation versus paracetamol in non-inflammatory rheumatic pain. *Rhumatologie - Reveu international de Rhumatologie* , 20 (1): 41-7.
- Brouwer RW, Jakma TS, Verhagen AP et al. (2005) Braces and orthoses for treating osteoarthritis of the knee. *Cochrane Database of Systematic Reviews* (1): CD004020.
- Carle (1992). Nabumetone compared with indomethacin in the treatment of osteoarthritis in general practice. *J Rheumatol* Suppl. 36:58-62.
- BCFI (2005). Intra-articulaire medicatie bij gonartrose. *Folia Pharmacotherapeutica*, 32, pp.23-4
- BCFI (2009). Antitrombotische behandeling: enkele recente publicaties. *Folia Pharmacotherapeutica*, 36, pp.10-4
- BCFI (2011). Geneesmiddelenbewaking: levertoxiciteit van paracetamol aan therapeutische dosis bij risicopatiënten. *Folia Pharmacotherapeutica*, 38, p.36

- BCFI (2012). Terugbetaling van paracetamol bij chronische pijn. *Folia Pharmacotherapeutica*, 39, pp.77-78
- BCFI (2013). Gecommentarieerd Geneesmiddelenrepertorium. www.bcfi.be.
- CBO (2007). Richtlijn diagnostiek en behandeling van heup- en knieartrose.
- Cepeda MS, F. C. (2006). Tramadol for Osteoarthritis. *Cochrane Database for Systematic Reviews* , Issue 3, Art. No.: CD005522. DOI: 10.1002/14651858.CD005522.pub2.
- Chen Y-F, J. P. (2008). Cyclooxygenase-2 selective non-steroidal anti-inflammatory drugs for osteoarthritis and rheumatoid arthritis: a systematic review and economic evaluation. . *Health Technol Assess* , 12 (11).
- Chevalier P. (2008). Cardiovasculair risico van celecoxib. *Minerva* , volume 7 (8): 120-121.
- Chou R, M. M. (2011). Analgesics for Osteoarthritis: An Update of the 2006 Comparative Effectiveness Review. . *Agency for Healthcare Research and Quality*.
- Christensen R, Bartels EM, Astrup A et al. (2007) Effect of weight reduction in obese patients diagnosed with knee osteoarthritis: a systematic review and meta-analysis. *Annals of the Rheumatic Diseases* 66 (4): 433–9.
- CKS. (2008, last update 2010). Guideline osteoarthritis. http://www.cks.nhs.uk.

farmaka

- Clegg DO, R. D. (2006). Glucosamine, chondroitin sulfate, and the two in combination for painful knee osteoarthritis. GAIT-trial. *New Engl Journal of Medicine* , 354:795-808.
- Dalgin P. (1997). Comparison of tramadol and ibuprofen for the chronic pain of osteoarthritis. *Arthritis Rheum* , 40 suppl: S86.
- Divine JG, Z. B. (2007). Viscosupplementation for knee osteoarthritis: a systematic review. *Clin Orthop Relat Res* , 455:113-22.
- EMA (2012). Vig-news. Agende Fédérale des Médicaments et des Produits de Santé. 17.01.2013
- EULAR recommendations 2003: an evidence based approach to the management of knee osteoarthritis.
- EULAR evidence based recommendations 2007 for the management of hand osteoarthritis: report of a task force of the EULAR Standing Committee for International Clinical Studies Including Therapeutic (ESCISIT).
- Fleischmann (1992). Clinical efficacy and safety of nabumetone in rheumatoid arthritis and osteoarthritis. *RheumatolSuppl* , 36:32-40
- Fosbol EL, e. a. (2009). Risk of myo-cardial infarction and death associated with the use of nonsteroidal anti-inflammatory drugs (Nsaid’s) among healthy individuals: a nationwide cohort study. *Clin Pharmacol Ther* , 85:190-97.

farmaka

- Garcia Rodrigues LA, e. a. (2008). NSAID Use Selectively Increases the Risk of Non-Fatal Myocardial Infarction: A Systematic Review of Randomised Trials and Observational Studies. *J Am Coll* , 52: 1628-36.
- Gonzalez ELM, P. P. (2010). Variability Among Nonsteroidal Antiinflammatory Drugs in Risk of Upper Gastrointestinal Bleeding. *Arthritis & Rheumatism* , 62: 1592-1601.
- Henry D, L. L.-Y. (1996). Variability in risk of gastrointestinal complications with individual non-steroidal anti-inflammatory drugs: results of a collaborative meta-analysis. *BMJ* , 312:1563-6.
- Hippisley Cox J, C. C. (2005). Risk of myocardial infarction in patients taking cyclo-oxygenase-2 inhibitors or conventional non-steriodal anti-inflammatory drugs: population based nested case-control analysis . *BMJ* , 330:1366.
- RIZIV (2010) http://www.riziv.fgov.be/drug/nl/drugs/recommendation/pdf/recommandations_PPI_IPP.pdf
- Kjaersgaard AP, N. A. (1990). Codeine plus paracetamol versus paracetamol in longer-term treatment of chronic pain due to osteoarthritis of the hip: A randomised, double-blind, multi-centre study. *Pain* , 43 (3): 309-18.

- Lin J, Z. W. (2004). Efficacy of topical non-steroidal anti-inflammatory drugs in the treatment of osteoarthritis: meta-analysis of randomised controlled trials. *BMJ* , 329:324-326.
- MacDonald (1997). Association of upper gastrointestinal toxicity of nonsteroidal antiinflammatory drugs with continued exposure: cohort study. *BMJ*. 315:1333-7
- Michels J, Demulder A, Dirven K, et al. Aanbeveling voor goede medische praktijkvoering: Orale Anticoagulatietherapie door de huisarts. Huisarts Nu 2010;39:S1-S36.
- Morgan (1993). Efficacy and safety of nabumetone versus diclofenac, naproxen, ibuprofen, and piroxicam in the elderly. *Am J Med*., 95(2A):19S-27S
- MRS, formulaire, 2012.
- NICE. (2008). Osteoarthritis National clinical guideline for care and management in adults.
- Nüesch E, R. A. (2009). Oral or transdermal opioids for osteoarthritis of the knee of hip. *Cochrane Database of Systematic Reviews* .
- Pavelka K, P. Z. (1998). Intraindividual differences in pain relief and functional improvement in osteoarthritis with diclofenac or tramadol. *Clinical Drug Investigation* , 16(6):421-9.
- Prescrire (1999). Nabumétone: un ains sans originalité. *La Revue Prescrire* (19) nr 200: p729-732.

farmaka

- Prescrire (2011). Suppléments Interactions Médicamenteuses, 30 (326)
- Prieto (1997). Tolerability to nabumetone and meloxicam in patients with nonsteroidal anti-inflammatory drug intolerance. *J Allergy ClinImmunol*. 119(4):960-4.
- Rahme E, P. D. (2002). Determinants and sequelae associated with utilization of acetaminophen versus traditional nonsteriodal anit-inflammatory drugs in elderly population. *Arthritis Rheum* , 46(11):3046-54.
- Rejeski WJ, Focht BC, Messier SP et al. (2002) Obese, older adults with knee osteoarthritis: weight loss, exercise, and quality of life. *Health Psychology* 21 (5): 419–26.
- Roddy E, Zhang W, Doherty M (2005). Aerobic walking or strengthening exercise for osteoarthritis of the knee? A systematic review. *Annals of the Rheumatic Diseases* 64 (4): 544–8.
- Silverstein FE, F. G. (2000). Gastrointestinal toxicity with celecoxib vs nonsteroidal anti-inflammatory drugs for osteoarthritis and rheumatoid arthritis: the CLASS study: A randomized controlled trial. Celecoxib Long-term Arthritis Safety Study. *JAMA* , 284(10): 1247-55.
- SSPF (2007). Utilisation concommitante d’ibuprofène et d’aspirine. *SSPF* janvier 2007
- Towheed. (2006). Acetaminophen for osteoarthritis (Review). *Cochrane Database of Systematic Reviews* . Issue 1. Art. No.: CD004257. DOI: 10.1002/14651858.CD004257.pub2.

- Towheed T. (2006). Pennsaid therapy for osteoarthritis of the knee: a systematic review and metaanalysis of randomized controlled trials. *J Rheumatol* , 33:567-573.
- Towheed TE, M. L. (2005). Glucosamine therapy for treating ostoeoarthritis. *Cochrane Database Syst Rev* , Issue 2. Art. No.: CD002946. DOI:10.1002/14651858.CD002946.pub2.
- Trelle S, R. S. (2011). Cardiovascular safety of non-steroidal anti-inflammatory drugs: a network meta-analysis. *BMJ* , 342:c7086, 1-11.
- Verduijn M.M., F. (2004). NHG standpunt: Wat is de maximale dagdosering van paracetamol voor langdurig gebruik bij benigne aandoeningen? *NHG* .
- Vignon (2006). Osteoarthritis of the knee and hip and activity: a systematic international review and synthesis. *Joint Bone Spine*, 73:442-55.
- Vlad (2007). Glucosamine for Pain in Osteoarthritis. Why Do Trial Results Differ? *Arthritis & Rheumatism*, 56: 2267–2277
- Zacher J, B. K. (2001). Originalarbeiten - Topisches Diclofenac-Emulgel versus orales Ibuprofen inder Therapie der aktivierten Arthrose der Fingergelenke (Heberden- und/oder Bouchard-Arthrose). *Aktuelle Rheumatologie* , 26:7-14.

farmaka

- Zhang W, D. M. (2007). EULAR evidence based recommendations for the management of hand osteoarthritis: report of a task force of the EULAR Standing Committee for International Clinical Studies Including Therapeutic (ESCISIT). *Ann Rheum Dis* , 66:377-388.

farmaka

farmaka

onafhankelijk centrum voor geneesmiddeleninformatie
centre indépendant d'information sur les médicaments
